# Poly(A) tail regulation in stem cells and early development

**DOI:** 10.1186/s13619-025-00250-0

**Published:** 2025-11-25

**Authors:** Xiaosu Miao, Guang Hu

**Affiliations:** Epigenetics and RNA Biology Laboratory, National Institute of Environmental Health Sciences, Research Triangle Park, Durham, NC 27709 USA

**Keywords:** Poly(A) tail, Polyadenylation, Deadenylation, Post-transcriptional regulation, Stem cells, Embryonic development

## Abstract

Eukaryotic mRNAs are polyadenylated at their 3’-ends, and the poly(A) tails play critical roles in post-transcriptional gene regulation by influencing mRNA stability and translation. Here, we describe the biological processes and major protein factors that control poly(A) tail synthesis and shortening. We also discuss recent breakthroughs in poly(A) tail sequencing methods that enable high throughput and accurate measurement of poly(A) tail lengths. Finally, we review how poly(A)-tail regulators and poly(A)-tail-mediated post-transcriptional mechanisms affect stem cell fate and early embryonic development.

## Background

In eukaryotes, newly synthesized transcripts are polyadenylated in their 3’-ends to become mature messenger RNAs (mRNAs), adopting poly(A)-tails ranging from tens to hundreds of nucleotides in length. Poly(A) tail is added co-transcriptionally in the nucleus during 3’-end processing via two consecutive enzymatic steps: endonucleolytic RNA cleavage and homopolymeric tail synthesis (Zhao et al. 1999; Edmonds 2002). Poly(A) tail is required for mRNA export from the nucleus, and its length is subjected to additional modifications in the cytosol via polyadenylation and deadenylation. Poly(A) tail influences both the stability and translational efficiency of mRNAs, and thereby plays a critical role in post-transcriptional gene regulation (Fuke and Ohno 2008). Consistently, genes involved in poly(A) tail regulation have been implicated in various developmental processes and diseases.

Here, we review the molecular steps involved in mRNA poly(A) tail synthesis and degradation, focusing on factors and processes that regulate poly(A) tail length dynamics. We also highlight recent advances in methodologies for poly(A) tail length measurement. Finally, we summarize and discuss the involvement of poly(A) tail length regulation in stem cell fate and early development.

## Regulation of poly(A) tail length

### mRNA 3’-end processing and polyadenylation

The presence of a non-DNA-templated poly(A) tail at the 3’-end of eukaryotic mRNAs was discovered more than 50 years ago (Darnell et al. 1971; Edmonds et al. 1971; Lee et al. 1971). Since then, it has been shown that the poly(A) tail is added during mRNA 3'-end processing in a two-step reaction: cleavage and polyadenylation. In the first step, the cleavage site is defined between an upstream polyadenylation signal (PAS), most frequently an AAUAAA hexamer, and a GU-rich downstream element (DSE) by two evolutionarily conserved multi-subunit protein complexes CPSF (cleavage and polyadenylation specificity factor) and CstF (cleavage stimulation factor). In mammals, the CPSF complex is composed of two modules: the mammalian polyadenylation specificity factor (mPSF) and mammalian cleavage factor (mCF) module. mPSF contains four subunits: CPSF160, CPSF30, WDR33, and hFIP1. CPSF30 and WDR33 recognize the PAS in the nascent RNA, and CPSF160 and hFIP1 recruit the poly(A) polymerase (PAP) to catalyze polyadenylation. mCF contains three subunits: CPSF73, CPSF100 and SYMPLEKIN, with CPSF73 being the endonuclease that catalyzes the cleavage reaction approximately 20 nucleotides downstream of PAS. In addition to CPSF, the CstF complex is also required for cleavage. It contains three subunits: CstF50, CstF64, and CstF77, and recognizes the DSE in RNA (Clerici et al. 2017). The cleavage reaction releases the pre-mRNA from RNA polymerase II and creates a free 3’-end. It also defines the 3’-end of the mature transcripts and the C-terminal sequence of the 3′-untranslated regions (3’-UTRs) (Rodriguez-Molina and Turtola 2023). In the second step, the newly formed 3’-end of the pre-mRNA becomes the substrate of PAP, a template-independent RNA polymerase (Martin et al. 2000), and poly-adenosines are added in an untemplated manner.

The cleavage step in 3’-end processing is mediated by numerous factors and is highly conserved across eukaryotes. It has been extensively studied and the regulators involved are becoming increasingly well-defined. In comparison, the polyadenylation step appears to differ between yeast and higher eukaryotes, and the underlying molecular details are not fully understood. One important question is: in the absence of a template, how are the synthesis, elongation, and termination of a poly(A) tail regulated? One important player involved in these processes is the poly(A)-binding protein (PABP). There are multiple PABPs in mammals, which can be categorized into two groups based on their subcellular localization and structure (Kuhn and Wahle 2004). One is nuclear PABPs, including PABPN1, PABPN1L, and ZC3H14; and the other is cytoplasmic PABPs, including PABPC1, PABPC1L, PABPC3, PABPC4, PABPC4L, and PABPC5. PABPs bind poly(A)-tails with high affinity and a footprint of approximately 25 nucleotides. They can form oligomers along the poly(A)-tail, potentially serving as a counting mechanism for cells to sense tail length. Furthermore, they can interact with and recruit other proteins, such as the translation machinery and deadenylases, to regulate poly(A)-tail dynamics and mRNA fate.

In mammalian cells, poly(A) tail is thought to be synthesized to a relatively uniform length in the nucleus, a process governed by complex interactions across PAP, CPSF, and PABPN1. Based on early biochemical experiments, CPSF binds to the polyadenylation signal in pre-mRNAs, recruits PAP, and stimulates PAP activity to initiate poly(A) synthesis (Hamilton et al. 2019). PABPN1 then binds to the nascent poly(A) tails, where it cooperatively stimulates PAP, along with CPSF, to add more adenosines to elongate the poly(A) tails (Kühn et al. 2017). However, once the poly(A) tail reaches a length of approximately 250 nucleotides (nt), PABPN1 disrupts the stimulation of PAP by CPSF, thus terminating processive polyadenylation (Kühn et al. 2009). In essence, PABPN1 oligomerizes on the growing poly(A) tail and acts as a molecular ruler to modulate CPSF-stimulated PAP activity. In support of this model, a recent study using transcriptome-wide long-read sequencing showed that newly synthesized transcripts indeed have long poly(A) tails ranging from 200 to 250 nt (Alles et al. 2023). But many other details of this model remain to be investigated, such as the structural and biochemical properties of the involved proteins, as well as the genetic impact of their depletions on nascent poly(A) tail length. Moreover, whether and how other poly(A)-binding proteins are involved in poly(A) tail regulation has not been systematically examined.

After export to the cytoplasm, mRNAs in some cells can undergo additional polyadenylation, which serves as an important mechanism of post-transcriptional gene regulation. For example, cytoplasmic polyadenylation promotes maternal mRNA translational activation in oocytes and early embryos (Rouhana et al. 2023). Interestingly, some nuclear mRNA polyadenylation factors are also responsible for cytoplasmic polyadenylation (Charlesworth et al. 2013). In addition, non-canonical poly(A) polymerases and terminal uridylyl-transferases can extend poly(A) tails and add non-adenosine residues, further modulating mRNA stability and translation efficiency (Yu and Kim 2020; Liudkovska and Dziembowski 2021; Lee et al. 2024; Liu and Lu 2024).

### Deadenylation

After their initial synthesis, poly(A) tails undergo significant length editing throughout the life cycle of mRNAs. As mentioned above, nascent poly(A) tails have lengths in the range of 200~250 nt. Using metabolic labeling and cellular fractionation, recent studies showed that poly(A) tails are quickly shortened both before and after their export to the cytosol and reach a steady-state length of 100~150 nt (Eisen et al. 2020; Liu et al. 2023a, b, c). Thus, steady-state poly(A)-tails are intrinsically heterogenous, reflecting the presence of RNAs at different stages of their life cycle.

The shortening of the poly(A) tail, or deadenylation, is catalyzed by deadenylases and their co-factors. Deadenylation is the rate-limiting step in mRNA decay, as complete removal of the tail triggers de-capping and degradation of the mRNA from both 5’- and 3’-ends (Parker 2012). The deadenylases belong to an expanding family of 3’−5’ exoribonucleases, and there are three major deadenylase complexes in eukaryotes: PAN2-PAN3, CCR4-NOT, and PARN (poly(A)-specific ribonuclease) (Goldstrohm and Wickens 2008, Webster et al. 2018). Although they all perform the same biochemical reaction, they are not functionally redundant. PARN appears to be only found in vertebrates and may have specialized roles in small RNA biogenesis. PAN2-PAN3 and CCR4-NOT, on the other hand, account for the major cytoplasmic deadenylation activities in most cell types. Earlier studies showed that PAN2-PAN3 and CCR4-NOT act predominantly at different stages of mammalian poly(A) tail removal. PAN2-PAN3 is important for the initial phase of deadenylation, removing the distal part of the poly(A) tail (Yamashita et al. 2005). CCR4-NOT is involved in the second phase to remove adenosines that are more proximal to the 3’-UTR (Yi et al. 2018). Because mRNA decay occurs when the poly(A) tail becomes very short, this biphasic deadenylation model predicts that CCR4-NOT would have a more important role in mRNA stability than PAN2-PAN3 (Liu et al. 2022). Consistent with that notion, depletion of CCR4-NOT subunits, but not PAN2-PAN3, substantially elongated mRNA half-life and upregulated steady-state levels (Yi et al. 2018; Mostafa et al. 2020). The presence of multiple deadenylase complexes and the sequential deadenylation suggest that poly(A) tail length likely plays critical roles in RNA metabolism and post-transcriptional gene regulation. It will be interesting to further investigate whether and how different deadenylase complexes and subunits regulate poly(A) tail and RNA fate in different biological contexts.

In eukaryotes, CCR4-NOT appears to be the predominant deadenylase based on genetic and genomic experiments (Collart 2016; Yi et al. 2018). In mammals, CCR4-NOT is composed of 12 core subunits: CNOT1, CNOT2, CNOT3, CNOT4, CNOT6, CNOT6L, CNOT7, CNOT8, CNOT9, CNOT10, CNOT11, TNKS1BP1 (Raisch and Valkov 2022). CNOT1 is the largest subunit and acts as a scaffold. CNOT2 and CNOT3 form a heterotrimer with the C-terminus of CNOT1 to stabilize the complex and serve as an interface for CCR4-NOT interacting proteins. CNOT9 binds the central region of CNOT1 and regulates the activity of the complex. CNOT10 and CNOT11 binds to the N-terminus of CNOT1 and form another module for protein–protein interactions. Notably, multiple CCR4-NOT subunits possess deadenylase activity, including CNOT6, CNOT6L, CNOT7, and CNOT8. However, only one subunit from the CNOT6-CNOT6L pair and one from the CNOT7-CNOT8 pair can be incorporated into the same complex (Winkler and Balacco 2013; Stoney et al. 2022). As a result, CCR4-NOT likely exists as a heterogeneous mixture with different deadenylase combinations in vivo. The CCR4-NOT deadenylase subunits have partially overlapping functions, but also exhibit tissue- and cell-type-specific roles and expression patterns. In mice, deletion of various enzymatic subunits led to distinct phenotypical outcomes: *Cnot4* deletion caused male infertility (Dai et al. 2021a, b); *Cnot6* deletion had no obvious phenotypes (Mostafa et al. 2020), while *Cnot6l* deletion led to metabolic abnormalities (Dai et al. 2021a, b) and *Cnot6*/*Cnot6l* double deletion resulted in female infertility (Dai et al. 2021a, b); *Cnot7* deletion also caused male infertility (Berthet et al. 2004); and *Cnot8* deletion led to early embryonic lethality (Mostafa et al. 2020). Moreover, CNOT6 and CNOT7 were found to display different substrate preferences and activities in vitro, depending on whether poly(A) tails were free or bound by poly(A)-binding protein (Webster et al. 2018, Yi et al. 2018). It will be interesting to further test whether and how the different subunits affect the substrate specificity and activity of the complex in vivo.

Intriguingly, the deadenylation rates of different mRNAs can vary by more than 1000-fold (Eisen et al. 2020).While mRNA secondary structure is one determinant of this variability (Stowell et al. 2016; Mauger et al. 2019), the leading model suggests that cis-regulatory sequences in mRNAs, often located in the 3’-UTRs, are responsible for transcript-specific deadenylation. These *cis*-regulatory sequences are recognized by RNA-binding proteins (RBPs) or microRNAs, which in turn recruit deadenylase complexes to target the corresponding mRNAs. For example, it is well-established that CCR4-NOT can be recruited to target mRNAs via RBPs such as the TTP, Pumilio/FBF, and Nanos family proteins to regulate gene expression (Mayya and Duchaine 2019). In addition, the RNA base modification m6A is another important player. It can directly recruit CCR4-NOT via the m6A reader YTHDF2 to deadenylate and degrade target transcripts (Collart 2016; Du et al. 2016). It can also alter RNA secondary structures or modify RBP and miRNA binding sites to indirectly influence the recruitment of deadenylase complexes (Lee et al. 2020).

## Poly(A) tail in post-transcriptional gene regulation

The poly(A) tail is essential for mRNA maturation and export from the nucleolus to the cytoplasm. In addition, the length of poly(A) tail and its dynamic changes play crucial roles in various aspects of mRNA metabolism, influencing post-transcriptional gene expression. Deadenylation is the first step in mRNA decay (Yamashita et al. 2005; Zheng et al. 2008), as it exposes the 3’-end of the mRNA to RNA exosome nucleases. Deadenylation is also closely coupled with de-capping, because the 5’- and 3’-ends of mRNAs typically form a closed loop via eIF proteins to prevent the association of de-capping enzymes (Vicens et al. 2018). Counterintuitively, the steady-state poly(A) tail length does not directly dictate transcript stability. Instead, the initial cytoplasmic tail length, which serves as an approximation for deadenylation rate, correlates well with mRNA half-life (Eisen et al. 2020). In fact, mathematical modeling showed that mRNA deadenylation and degradation rates are coherently regulated, such that the faster an mRNA is deadenylated, the more rapidly it is cleared from the cell. Additional investigations are needed to further understand the mechanism that governs the deadenylation rate, steady-state poly(A)-tail length, and mRNA stability.

In addition to its role in transcript stability, poly(A) tail length is also well-documented to influence mRNA translation. Generally, it is thought that mRNAs with short poly(A) tails (for example < 10As in human cells (Biziaev et al. 2024)) are poorly translated, while mRNAs with increasing poly(A) tail lengths acquire higher translation efficiency (Xiang and Bartel 2021). As the tails become long enough, the translation efficiency eventually reaches a plateau (Passmore and Coller 2022a). Recent studies showed that short poly(A) tails are associated with highly expressed genes across eukaryotes (Lima et al. 2017). These observations can be largely explained by the “closed loop” model of translation regulation (Biziaev et al. 2024). In this model, the mRNA 5’-cap is bound by the eukaryotic translation initiation factor 4E (eIF4E), while the 3’-poly(A) is bound by the cytoplasmic poly(A)-binding protein PABPC (Vicens et al. 2018). Both eIF4E and PABPC interact with another initiation factor eIF4G, which brings the RNA into a closed-loop structure: 5’-cap-eIF4E-eIF4G-PABPC-3’-poly(A) (Wells et al. 1998). This conformational arrangement significantly promotes translation initiation, at least in part, by recruiting the ribosome (Kahvejian et al. 2005). Intriguingly, while poly(A) tail length was found to be coupled to translational efficiency in oocytes and early embryos, this coupling disappeared in late or non-embryonic cells (Subtelny et al. 2014; Yang et al. 2017; Xiong et al. 2022). Thus, poly(A) tail likely regulates translation in a dynamic and context-dependent fashion.

## Approaches to determine poly(A) tail length

### Single gene-based methods

The growing interest in poly(A) tail length dynamics has created a pressing demand for accurate and high-throughput approaches to measure these tails. Conventional methods, such as the RNaseH/oligo-dT assay (Sippel et al. 1974), RACE-PAT (rapid amplification of cDNA ends poly(A) test) (Salles and Strickland 1995), LM-PAT (ligase-mediated poly(A) test) (Sallés et al. 1999), ePAT (extension poly(A) test) (Jänicke et al. 2012), sPAT (splint-mediated poly(A) test) (Minasaki et al. 2014), and HIRE-PAT (high-resolution poly(A) test) (Bazzini et al. 2012), significantly contributed to poly(A) tail studies in the early days and are still frequently used to validate newer methods (Brouze et al. 2023). However, they cannot provide precise poly(A) tail length estimation or composition and are often restricted to single-transcript analysis. To improve throughput, RNA fractionation using poly(U) or oligo-dT followed by detection of individual transcripts using microarray was employed to simultaneously assess poly(A) tails of all detectable transcripts (Salles and Strickland 1995). But fractionation cannot be used to determine absolute tail lengths or separate RNA with subtle to moderate changes in tail lengths. With advances in high-throughput sequencing (HTS) technologies, HTS-based approaches have quickly evolved in recent years to overcome previous constraints and provide a more comprehensive and systematic view of poly(A) tail dynamics.

### High-throughput sequencing (HTS)-based methods

The first two HTS-based poly(A) sequencing methods, PAL-seq and TAIL-seq, were developed in 2014 using the Illumina sequencing platform. Both methods rely on similar experimental steps to capture and amplify intact mRNA 3’-ends with poly(A) tails but employ different strategies to bypass the challenge with Illumina sequencing on homopolymers. PAL-seq used a customized oligo-dT hybridization approach to estimate poly(A) length, while TAIL-seq developed an algorithm to determine poly(A) length from raw sequencing data at single-nucleotide resolution (Chang et al. 2014; Subtelny et al. 2014). In the following years, TAIL-seq was further optimized into mTAIL-seq (Lim et al. 2016), and PAL-seq incorporated some concepts of TAIL-seq and became PAL-seq v2/v3/v4 (Eisen et al. 2020). These poly(A)-sequencing methods provided high-resolution and transcriptomic level analysis on poly(A) tails for the first time, dramatically expanding our knowledge about this important but difficult-to-study feature in mRNAs. Equipped with the new tools, many intriguing facts about mRNA 3’-ends were quickly uncovered, reshaping the view of poly(A) tail length and its mechanistic role in RNA metabolism. For example, it was found that poly(A) tails are shorter than previously considered in mammalian cells (Chang et al. 2014; Subtelny et al. 2014). The correlation between poly(A) length and translation efficiency is context dependent (Subtelny et al. 2014; Yang et al. 2017; Passmore and Coller 2022a; Xiong et al. 2022). mRNA 3’-ends contain wide-spread non-adenosine residues and RNA modifications to regulate their stability (Lim et al. 2014; Lim et al. 2018, Viegas, de Macedo et al. 2022). Finally, the deadenylation rate, but not the steady-state poly(A) tail length, dictates mRNA decay (Eisen et al. 2020).

In addition to PAL-seq and TAIL-seq, other methods using different molecular biology procedures to capture and amplify poly(A)-tails were developed, including PAT-seq (Bell et al. 2016), TED-seq (Woo et al. 2018), circTAIL-seq (Gazestani et al. 2016), and Poly(A)-seq (Yu et al. 2020). All these approaches provide nucleotide- or near-nucleotide-resolution poly(A) tail length measurements at a transcriptomic scale, greatly facilitating the research on poly(A) tail in post-transcriptional gene regulation. However, there are clear limitations due to the intrinsic properties of the poly(A) tails and the Illumina platform. First and foremost, the PCR amplification steps used in sequencing library preparations are known to introduce strong biases against longer poly(A) tails, as homopolymers are notoriously difficult to amplify. For the same reason, the cluster generation step during Illumina sequencing may exacerbate the bias even further. Indeed, there are often accumulations of reads with very short poly(A) tails (0~20 nt) in data generated by the above methods, which are likely amplification artifacts and may obscure the underlying biology. Second, Illumina sequencing only produces short read lengths (typically up to 300 bp), making it difficult or impossible to measure longer tails. Third, analysis of Illumina data on poly(A) tails presents significant bioinformatic challenges due to errors and missed calls associated with homopolymer sequencing. Fourth, most of the above methods require a significant amount of input RNAs and do not apply to studies with constraints on starting materials. Finally, results from different methods appear to show limited correlation and consistency, suggesting that the accuracy and reproducibility of these methods need further optimization.

Pacific BioSciences (PacBio) offers long-read sequencing (up to 25 kb) with very high accuracy. As a result, it handles homopolymers such as poly(A) tails much better than Illumina. Taking advantage of that, FLEP-seq and FLEP-seq2 (Long et al. 2021; Jia et al. 2022), FLAM-seq (Legnini et al. 2019), PAIso-seq and PAIso-seq2 (with optimizations to detect internal and 3′-end non-A residues) (Liu et al. 2019; Liu et al. 2023a, b, c), and SM-PAT-seq (Coon et al. 2021) were developed for poly(A) tail sequencing on the PacBio platform (Wenger et al. 2019). They vary slightly in sequencing library preparation but rely on similar conceptual approaches to capture, amplify, and sequence full-length mRNA with poly(A) tails. Impressively, PAIso-seq has been successfully applied to study transcriptome-wide poly(A) tails in mammalian oocytes and early embryos (Liu et al. 2023a, b, c), demonstrating both the importance of poly(A)-tail-mediated post-transcription mechanisms in gene regulation in development and the power of sequencing-based approaches in poly(A)-tail measurement. It is worth noting that while these PacBio-based methods appear to better measure long poly(A) tails, they still rely on PCR amplifications and therefore face the same challenge in preserving mRNA poly(A) tail length profiles during library preparation. In addition, PacBio sequencing is more costly and is not as commonly accessible in comparison to the Illumina platform, limiting its adoption in poly(A) tail studies.

The latest advancements in poly(A)-tail measurement are developed by Oxford Nanopore Technologies (ONT). ONT offers direct RNA sequencing (DRS), which, as the name indicates, sequences mRNAs directly without amplification, avoiding biases in tail-length profiles introduced by PCR (Garalde et al. 2018). It generates long reads up to hundreds of kilobases, enabling real-time sequencing of individual RNAs at full length. Poly(A)-tail length determination by DRS does not rely on base-calling and is therefore not limited by read lengths or qualities. Instead, it is estimated at a single-nucleotide resolution based on the duration it takes for the poly(A) tail to pass through the nanopore on the flow cell. In addition to measuring poly(A) tails, ONT DRS also provides information on differential expression, splicing, and RNA modifications. In addition to DRS, ONT also offers a cDNA-PCR sequencing kit that captures intact poly(A)-tails during cDNA reverse transcription, and then PCR amplifies the cDNAs for sequencing. However, as mentioned above, the PCR step likely introduces biases against long poly(A)-tails, making the cDNA-PCR protocol a less optimal approach. A better method called Nano3P-seq was recently developed to examine poly(A)-tail length by directly sequencing the first-strand cDNA without amplification. It uses a 3’-end-capture procedure that is similar to DRS and cDNA-PCR to preserve poly(A)-tail length during reverse transcription and allows more faithful determination of tail lengths. These strategies make ONT an increasingly popular tool for studying RNAs, especially the poly(A) tails. Apart from the strengths, there are still several disadvantages to using ONT DRS, including lower throughput and higher cost per read compared to Illumina, high error rates, the requirement for large amounts of starting material, and under-developed data analysis pipelines.

Although poly(A) tail measurement has been a significant development in recent years, further improvements and optimizations are still needed. It was reported that many current techniques do not produce consistent measurements of poly(A) tail length when cross-compared to each other, likely due to differences in sample preparations and sequencing platforms (Brouze et al. 2023). In addition, most methods still rely on PCR-based amplification, which is known to cause bias. Finally, the sample preparation and sequencing cost is still relatively high. Future innovations in poly(A) tail sequencing will need to overcome these challenges to facilitate the study of poly(A) tail biology.

## Poly(A) tail regulation in stem cells and early development

The poly(A) tail plays an essential role in post-transcriptional gene regulation by influencing mRNA stability and/or translation. Consequently, regulatory mechanisms that control poly(A) tails are pivotal in various biological processes, including cell signaling, growth, fate determination and fate transitions. Indeed, poly(A) binding proteins, deadenylases, and their interacting RNA-binding proteins (RBPs) have been implicated and in some cases extensively studied in DNA damage response and genomic integrity, stem cell maintenance, germ cell development, embryogenesis, lymphocyte differentiation and selection, heart function, bone aging, neural development, and neurodegeneration (Liu et al. 2023a, b, c). Below we will focus on the roles of deadenylation and deadenylation complexes in stem cells and early embryonic development.

### Poly(A) tail regulation in stem cells

Stem cells can both self-renew to maintain their population and differentiate into specialized cell types to support development. They are essential for organism development and the maintenance of homeostasis. While earlier studies primarily focused on signaling and transcriptional control in stem cell fate choices, recent research has highlighted the essential roles of poly(A) tail-mediated post-transcriptional regulation in stem cell maintenance and differentiation (Chen and Hu 2017).

The earliest documented cases of poly(A) tail involvement in stem cell fate regulation came from studies of the germline. Across species from invertebrates to mammals, many RBPs, including PUF and NANOS family proteins and DND1, are required for germline stem cell self-renewal (Shigunov and Dallagiovanna 2015; Mercer et al. 2021). They bind specific sequence motifs in the 3’-UTRs of target transcripts and recruit CCR4-NOT to repress their expression. Consistent with this, *Cnot3* and *Cnot7* in CCR4-NOT are required for male spermatogenesis in mice, and *Cnot3* is required for spermatogonia stem cell maintenance (Dai et al. 2021a, b, Chen et al. 2023).

In addition to germ cells, poly(A) tail regulation is also crucial for pluripotent stem cells. Multiple CCR4-NOT subunits, including CNOT1, CNOT2, and CNOT3, were initially identified to be essential for mouse embryonic stem cell (ESC) maintenance in genome-wide RNAi screens (Zheng et al. 2012). They were later found to be required for human ESCs self-renewal as well. Furthermore, genomic studies suggested that CNOT3 is required for maintaining the pluripotent state via deadenylation and degradation of differentiation gene transcripts (Zheng et al. 2016). Beyond self-renewal, CCR4-NOT complex also regulates ESC pluripotency and differentiation. For example, CNOT3 interacts with the Aurora B and MAPK/ERK kinases to promote the survival of differentiating meso-endodermal progenitor cells during ESC differentiation (Sarkar et al. 2021). CNOT8 is required for the deadenylation and degradation of naïve pluripotency gene transcripts and the differentiation from the naïve to the formative pluripotent state (Quan et al. 2022). The CCR4-NOT-interacting protein RNF219 facilitates neuronal lineage commitment during ESC differentiation (Du et al. 2020). Together, these findings support the idea that different subunits of CCR4-NOT complex may influence how the complex interacts and regulates various mRNA substrates, allowing the complex to control the fate of pluripotent stem cells.

Apart from maintaining pluripotency in ESCs, mRNA deadenylation is also important for the reprogramming of somatic cells into induced pluripotent stem cells (iPSCs). It has been reported that both CCR4-NOT and PAN2-PAN3 complexes can be recruited by YTHDF2/3, likely to m6A-containing RNAs, to facilitate the clearance of somatic gene mRNAs, thereby promoting somatic reprogramming (Liu et al. 2020). Consistently, overexpression of deadenylase subunits in CCR4-NOT complex enhances iPSC generation and the conversion of partially reprogrammed cells toward fully reprogrammed iPSCs (Kamon et al. 2014; Zukeran et al. 2016).

Finally, CCR4-NOT has been studied in several other types of stem cells and progenitors. CNOT3 was found to be required for spermatogonia stem cell maintenance (Chen et al. 2023). It also promotes the degradation of anti-proliferation gene transcripts in cardiac progenitors during cardiomyocyte differentiation (Yamaguchi et al. 2018). It is required for mRNA degradation in lymphoid progenitors and facilitates pro-B-to-pre-B-cell differentiation (Akiyama and Yamamoto 2021). In planarians, CCR4-NOT drives stem cell differentiation and promotes stem cell-specific mRNA degradation in planarian neoblasts (Solana et al. 2013). Together, these findings demonstrate that poly(A) tail regulation via deadenylation plays a critical role in various stem cell functions. It will be interesting to further dissect how specific mRNA targets are selected by individual deadenylases to regulate different aspects of stem cell fate in different developmental contexts.

### Poly(A) tail regulation in embryonic development and oocytes

Given the importance of poly(A) tail regulation in stem cells, it is expected that poly(A) tail-mediated gene regulation plays a critical role in development. ESCs are thought to exist in a pluripotent state similar to and are the in vitro counterpart of the epiblast cells from developing blastocyst embryos. As a result, factors involved in ESC self-renewal or differentiation frequently affect early development. Indeed, CCR4-NOT is essential during peri-implantation development, as expected based on findings in ESCs. CNOT3 deletion leads to peri-implantation lethality and diapause defects, consistent with its role in pluripotency maintenance (Zheng et al. 2016). CNOT8 deletion results in embryonic lethality at the beginning of gastrulation, in agreement with its role in ESC differentiation (Soeda et al. 2023). CNOT9 null mice appear normal immediately post-gastrulation, but exhibit growth and differentiation defects accompanied by extensive cell death by embryonic day 9.5 (E9.5) (Sarmah et al. 2020). In addition to CCR4-NOT, deletion of the deadenylase PARN is embryonic lethal before E11.5, although the underlying mechanism is not clear (Benyelles et al. 2019). Deletion of the poly(A) tail binding protein PABPN1 also leads to failure in embryonic development, in line with its known role in governing the initial poly(A) tail length during synthesis (Zhao et al. 2021). Finally, non-A residues have been shown to be prevalent in matured oocytes and pre-implantation embryos, suggesting that poly(A)-tail composition is also important for post-transcriptional regulation (Liu et al. 2023a, b, c; Liu and Lu 2024). Together, these animal studies strongly argue that poly(A) tail is a key regulatory mechanism in embryonic development.

Poly(A) tail-mediated gene regulation seems to play an even bigger role in oocyte development and maternal-to-zygotic transition. In mammals, transcription rates are high during oocyte growth, and the developing oocytes generate significantly more mRNAs than what is typically found in somatic cells. As oocytes reach their mature size, transcription becomes inactive and post-transcriptional mechanisms take over to control the gene expression program (Li et al. 2010). Transcription remains largely silent during ovulation and fertilization, and abundant RNA synthesis begins again after zygotic genome activation (Schultz 1993; Li et al. 2010). As a result, mRNAs synthesized during oocyte growth are not always used immediately. Instead, some mRNAs are stored and employed during later stages of development, from oocyte maturation, fertilization, to the two-cell stage in embryo development (Ma et al. 2001). Given that poly(A) tails affect mRNA stability and translation, it naturally becomes an essential mechanism for gene regulation in oocytes and early embryos.

On the poly(A) synthesis side, the nuclear poly(A)-binding protein PABPN1 is required for poly(A) synthesis in oocytes and its deletion leads to primary ovarian insufficiency and female infertility (Dai et al. 2022, Zhao et al. 2022). The maternally expressed PABPN1-like (PABPN1L) protein is required for maternal mRNA degradation after meiosis resumption and during the maternal-to-zygotic transition (Zhao et al. 2020; Emori et al. 2024). In addition, the embryo-specific cytoplasmic poly(A)-binding protein PABPC1L (also called ePAB) protects mature poly(A) tails and is required for oocyte growth and the acquisition of meiotic competence (Guzeloglu-Kayisli et al. 2012). In addition to the canonical nucleus polyadenylation, poly(A) tail can also be elongated by cytoplasmic polyadenylation in oocytes and early embryos (Radford et al. 2008; Charlesworth et al. 2013; Rodriguez-Molina and Turtola 2023). This cytoplasmic polyadenylation activates the translation of dormant stored mRNAs and promotes oocyte maturation and maternal-to-zygotic transition (Radford et al. 2008; Reyes and Ross 2016). However, it is not fully understood how this process is regulated and how the target mRNAs are selected.

In addition to synthesis, the timely removal of mRNAs is also critical for development to properly proceed (Yu et al. 2016). In *Xenopus*, PARN is responsible for poly(A)-tail shortening. In mammals, CCR4-NOT has been extensively studied. CNOT6L was shown to be required for the deadenylation and degradation of a large subset of maternal mRNAs during oocyte maturation (Sha et al. 2018). CNOT6 is also expressed in cortical foci of oocytes to regulate deadenylation (Vieux and Clarke 2018). Another deadenylase subunit CNOT7, recruited by BTG4, can shorten poly(A) tails and facilitate maternal mRNA decay (Liu et al. 2016, Pasternak et al. 2016, Wu and Dean 2016, Yu et al. 2016, Liu et al. 2023a, b, c). However, deadenylation does not always lead to mRNA degradation. Poly(A) tail lengths were shown to be coupled to translational efficiency in early zebrafish and frog embryos (Subtelny et al. 2014). Consistent with that, CNOT7 and CNOT8 were found to suppress untimely translational activation of maternal mRNAs via deadenylation to ensure proper oocyte maturation (Soeda et al. 2023). Recently, a new family of proteins, MARTRE1-MATRE6, were identified to directly interact with CCR4-NOT and inhibit deadenylation. They are required to maintain the optimal poly(A)-tail length and mRNA translation efficiency in mouse zygotes (Yang et al. 2025). Finally, it was shown that maternal mRNA deadenylation is defective in in vitro matured mouse and human oocytes, contributing to increases in maternal RNA abundance (Liu et al. 2024).

Animal studies and genetic evidence demonstrate that the gene expression program is heavily regulated at the post-transcriptional level via poly(A)-tails during early development, from oocyte maturation to gastrulation. To further elucidate how tail length affects gene expression and influences cell fate, it will be interesting to systematically examine changes in poly(A)-tail length during development in both normal and mutant animals with deletions of poly(A)-tail regulators.

## Conclusions

**Fig. 1:**
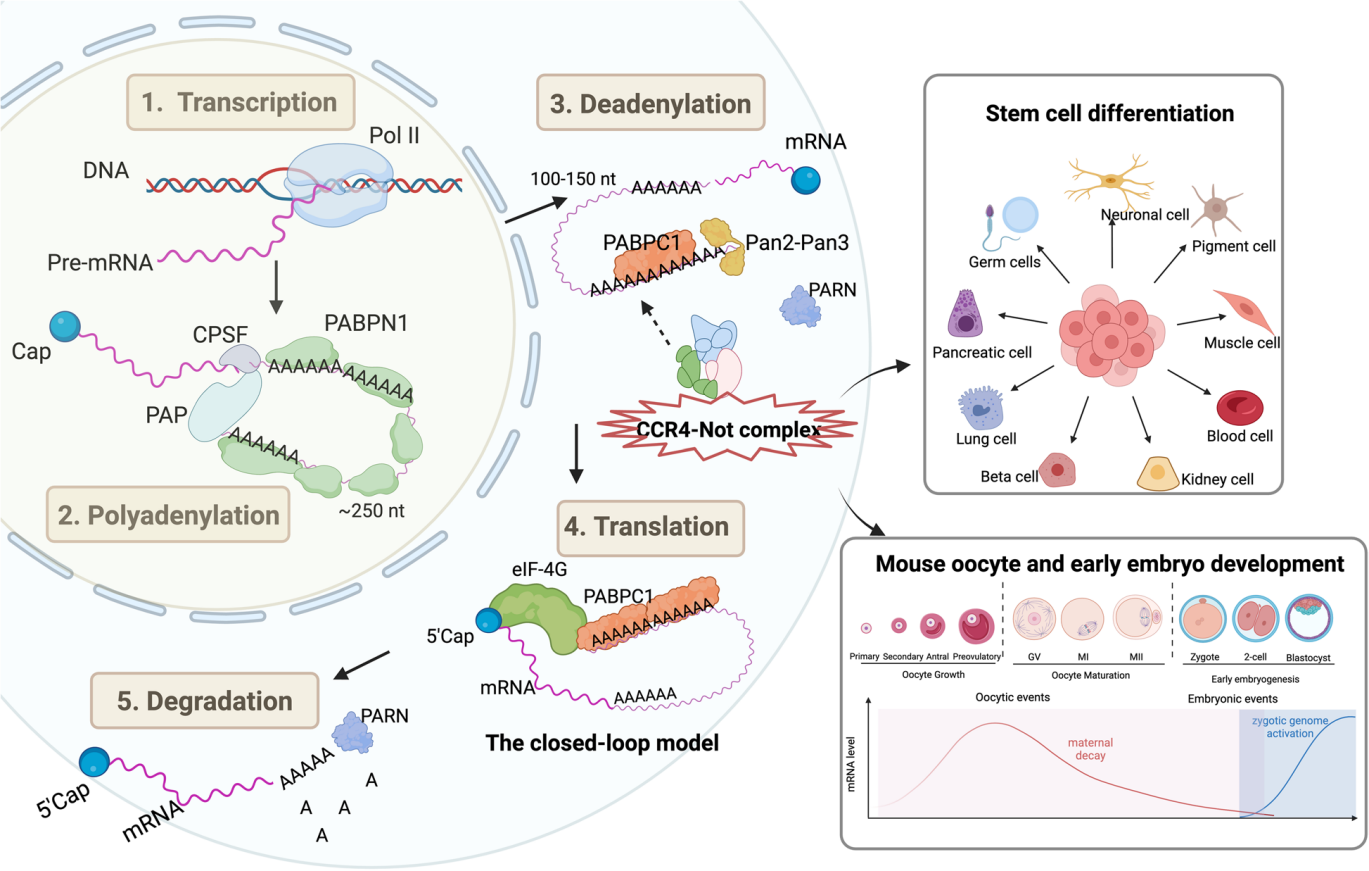
Regulation of Poly(A)-tails in stem cell and early development. Eukaryotic mRNAs are polyadenylated at their 3’-ends, generating the poly(A)-tails that play critical roles in mRNA stability and translation

Advances in genomic, biochemical, and structural biology methods have reinvigorated interest in poly(A)-tail biology. These innovations have enabled more accurate investigations into the absolute length and composition of poly(A) tails at the transcriptomic level, as well as the mechanisms of polyadenylation and deadenylation. Despite the progress discussed in this review (Fig. 1), many intriguing questions remain unanswered. For example, how is the steady-state poly(A)-tail length determined for individual transcripts? Why do different transcripts have different poly(A)-tail lengths? What are the roles of individual deadenylase complexes, and do they act independently or synergistically? What are the roles of individual subunits in each complex? Does and how does each deadenylase complex or deadenylase subunit regulate specific mRNA targets? How does poly(A)-tail length control specific developmental events? To answer these questions, it will be necessary to continually develop and optimize poly(A) tail sequencing methodologies. With advances in technologies, we hope to see a more comprehensive understanding of poly(A) tail-mediated post-transcriptional gene regulation across diverse biological systems shortly.

## Data Availability

Not applicable.

## Abbreviations

3’-UTRs: 3’-Untranslated regions
CPSF: Cleavage and polyadenylation specificity factor
CstF: Cleavage stimulation factor
DRS: Direct RNA sequencing
DSE: Downstream element
ePAT: Extension poly(A) test
ESC: Embryonic stem cell
HIRE-PAT: High-resolution poly(A) test
HTS: High-throughput sequencing
iPSCs: Induced pluripotent stem cells
LM-PAT: Ligase-mediated poly(A) test
mPSF: Mammalian polyadenylation specificity factor
mCF: Mammalian cleavage factor
ONT: Oxford Nanopore Technologies
PABP: Poly(A)-binding protein
PAP: Poly(A) polymerase
PARN: Poly(A)-specific ribonuclease
PAS: Polyadenylation signal
RACE-PAT: Rapid amplification of cDNA ends poly(A) test
RBPs: RNA-binding proteins
sPAT: Splint-mediated poly(A) test
